# Rabbit Hemorrhagic Disease Virus Detected in Pico, Azores, Portugal, Revealed a Unique Endemic Strain with More Than 17 Years of Independent Evolution

**DOI:** 10.3390/v6072698

**Published:** 2014-07-14

**Authors:** Pedro J. Esteves, Ana M. Lopes, Maria J. Magalhães, Ana Pinheiro, David Gonçalves, Joana Abrantes

**Affiliations:** 1 Centro de Investigação em Biodiversidade e Recursos Genéticos, InBIO Laboratório Associado (CIBIO), Vairão, 4485-661 Portugal; E-Mails: analopes@cibio.up.pt (A.M.L.); magalhaes.mjtr@hotmail.com (M.J.M.); ana.pinheiro@cibio.up.pt (A.P.); drgoncal@fc.up.pt (D.G.); jabrantes@cibio.up.pt (J.A.); 2 Departamento de Biologia, Faculdade de Ciências da Universidade do Porto, Porto, 4169-007, Portugal; 3 Instituto de Investigação e Formação Avançada em Ciências e Tecnologias da Saúde (CESPU), Gandra, 4585-116, Portugal; 4 INSERM, UMR892; CNRS, UMR629; Université de Nantes, Nantes, 44007, France; 5 SaBio—IREC (CSIC-UCLM-JCCM), Ciudad Real, 13071, Spain

**Keywords:** RHDV, European rabbit, Azores, endemism

## Abstract

Rabbit hemorrhagic disease is caused by a calicivirus, rabbit hemorrhagic disease virus (RHDV), which is responsible for high mortality in domestic and wild European rabbits (*Oryctolagus cuniculus*). RHDV strains were sequenced from wild European rabbits (*Oryctolagus cuniculus algirus*) collected in the Azorean island of Pico, Portugal. Phylogenetic analyses showed that the Pico RHDV strains diverge from all of the others described so far, but cluster with the genogroups 1–5 (G1–G5). The genetic distance between the Pico RHDV sequences and each G1, G2 and G3–G5 genogroup (~0.08) is compatible with an RHDV introduction at least 17 years ago. Our results show that in Pico, RHDV is the outcome of an independent evolution from the original RHDV strain that appeared in its European rabbit population. These are the first sequences of RHDV obtained in the subspecies *O. c. algirus*, outside of its original region, the Iberian Peninsula. Furthermore, we discuss the risk of rabbit translocations from the Azores to the Iberian Peninsula, where the rabbit wild populations are suffering high mortalities.

## 1. Introduction

In the Iberian Peninsula, two subspecies of European rabbit are found, *O. cuniculus* subsp. *algirus* and *O. cuniculus* subsp*. cuniculus*, both equally fatally susceptible to rabbit hemorrhagic disease virus (RHDV) [1]. The *O. c. algirus* subspecies is restricted to the southwest of the Iberian Peninsula, the Azores, Madeira and the Canary Islands. The introduction of the European rabbit in the Azores occurred in the 15th century by Portuguese navigators [2]. The genetic diversity of these populations is a subset of the genetic diversity of the Iberian Peninsula populations [3,4,5].

RHDV is a single-stranded, positive-sense RNA virus of the genus *Lagovirus*, family Caliciviridae, first detected in China in 1984, that then spread worldwide (reviewed in [1]). Genetic comparative studies showed that pathogenic and non-pathogenic RHDV strains are genetically independent, with more than 20% of divergence [6,7,8,9,10]. Pathogenic strains can be divided into three groups: RHDV, which include older strains of the genogroups, 1–5 (G1–G5) [11]; RHDVa (genogroup G6), characterized by a distinct antigenic profile [12]; and RHDV2, a new variant that differs from other pathogenic viral forms by more than 15% [13,14,15,16]. In the wild European rabbit populations from the Iberian Peninsula, all RHDV strains identified before 2011 belonged to G1 [17,18,19]. From 2012, only RHDV2 was detected, which suggests the replacement of G1 by RHDV2 [15,20,21]. In the Azores islands, the first outbreaks of RHDV were recorded from 1988–1993 [22,23,24]; the first reports of RHDV in Portugal date back to 1989 [1].

## 2. Materials and Methods

In 2013, our laboratory, Centro de Investigação em Biodiversidade e Recursos Genéticos CIBIO, Universidade do Porto, Portugal, received five rabbit liver samples, belonging to *O. cuniculus* subsp. *algirus*, collected in the Portuguese Azorean island of Pico. The rabbits appeared dead in the field and had gross (pathological) lesions compatible with rabbit hemorrhagic disease (RHD). Total RNA of the samples was extracted with the RNeasy Mini Kit (QIAGEN, Hilden, Germany), according to the manufacturer’s instructions. Reverse transcription (RT) was performed by using oligo(dT) as the primer (Invitrogen, Carlsbad, CA, USA) and SuperScript III reverse transcription (Invitrogen), as recommended by the manufacturer. Screening of the samples employed several RT-PCR assays designed to result in overlapping amplification products covering the entire VP60 gene (the PCR conditions are available on request). After purification, PCR products were sequenced on an automatic sequencer ABI PRISM 310 Genetic Analyzer (PE Applied Biosystems, Foster City, CA, USA) with the pairs of primers used for amplification.

All five samples were PCR-positive for RHDV. The obtained sequences were aligned with sequences available from public databases. Withdrawn sequences represent the RHDV groups G1–G5, RHDVa, RHDV2 and the nonpathogenic groups. Since the occurrence of recombination in the RHDV strains has been reported [25,26], we screened the alignment for recombination using RDP v4.35 [27]. The phylogenetic tree was inferred in MEGA6 [28] by using a maximum-likelihood (ML) approach. The reliability of the nodes was assessed with a bootstrap resampling procedure consisting of 500 replicates of the ML trees. The best-fit nucleotide substitution model was determined by using MEGA6. Pairwise genetic distances and between group means distances were calculated in MEGA6 [28].

## 3. Results

**Figure 1:**
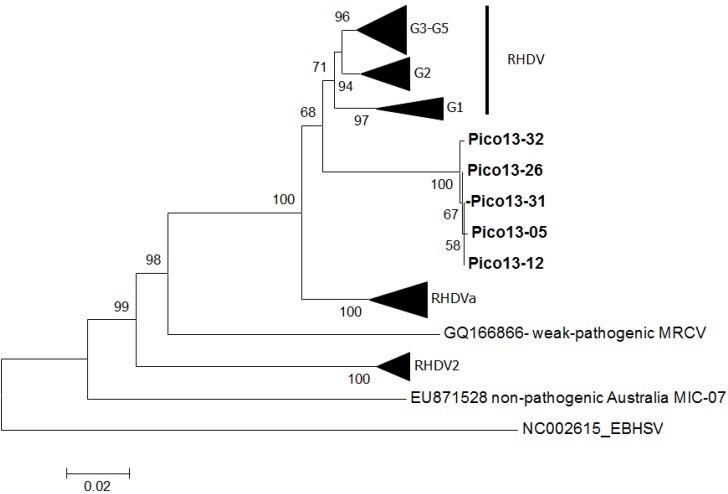
Maximum-likelihood phylogenetic tree of 74 complete capsid gene sequences of rabbit hemorrhagic disease virus (RHDV). Bootstrap values appear next to the nodes and are shown only for the major groups: RHDV Genogroup 1 (G1) (GenBank Accession Nos. JX886002, JF438967, JX886001, EF558578, Y15440, Z24757, L48547, Z49271, Z29514); RHDV G2 (GenBank Accession Nos. KF494932, KF494947, AF231353, KF494924, KF494921, U54983, EF558580, EF558579, M67473, EU003579, JN851735, EU650679, GU373618, AF402614, AF295785, FR823355, U49726, EU003580, FJ212323); RHDV G3-5 (GenBank Accession Nos. FR823354, AJ535092, EF558585, Y15441, EF558575, EF558577, Y15426, X87607, AJ535094, EF558574, Y15424, EF558573, EF558572); RHDV2 (GenBank Accession Nos. HE800529, HE800530, HE800531, HE800532, HE819400, FR819781, JQ929052, JX106023, KC345611, KC345612, KC345613); RHDVa (GenBank Accession Nos. EU003582, EF558583, EF558584, AB300693, EU003581, DQ205345, EF558582, AF258618, JF412629, HM623309, AY523410, EU003578, EF558581, DQ280493). European brown hare syndrome virus (EBHSV) was used to root the tree (GenBank Accession No. NC_002615). The non-pathogenic strain from Australia (GenBank Accession No. EU871528) and the moderate pathogenic strain from the United States of America (GenBank Accession No. GQ166866) were also included. The samples isolated from the rabbits found in Pico, Portugal, appear in bold (Pico13-05, Pico13-12, Pico13-26, Pico13-31, Pico13-32, GenBank Accession Nos. KJ579156–KJ579160). The scale bar indicates the nucleotide substitutions per site.

**Figure 2:**
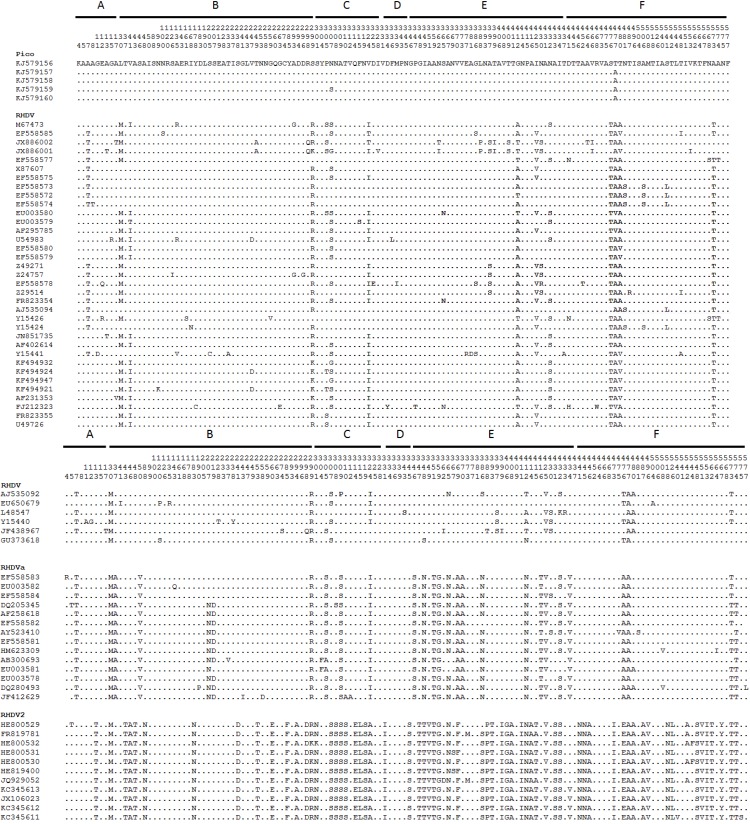
Amino acid polymorphic positions in the RHDV VP60 capsid. The different capsid regions are indicated at the top [29]. Dots represent the identity with the sequence Pico13-05 (GenBank Accession number KJ579156).

In the RHDV sequences recovered from Pico, a total of seven nucleotide changes were observed, two of which were non-synonymous. Genetic distances calculated using the pairwise distance between each of these sequences varied between 0.01% and 0.03%. The inferred ML phylogenetic tree is in agreement with those published (Figure 1) [14,15,16]. Indeed, the pathogenic strains appear in two very well-supported main groups: (1) RHDV strains (G1–G5) and RHDVa; and (2) the new variant, RHDV2. The Pico RHDV sequences (Pico-13) form a highly supported group (bootstrap value: 100), and although more closely related with the RHDV strains (G1–G5), they differ from each RHDV group (G1, G2 and G3–G5) by approximately 8% (between group means distances). No evidence of recombination was found. The amino acid differences obtained between Pico samples and the other pathogenic RHDV strains are shown in Figure 2. Six distinct regions (A to F) can be distinguished in the RHDV VP60 capsid. The C and E regions are located in the exposed P2 subdomain and show the highest degree of genetic variation [29]. The non-synonymous substitutions observed in Pico strains are localized in the region, C, amino acid 307, and F, amino acid 477 (Figure 2). Interestingly, positive selection for the amino acid position 307 has been reported; the position 477 is adjacent to residue 476, which has also been identified as evolving under positive selection [30].

## 4. Discussion

In the Iberian Peninsula, RHDV was first detected in 1988 in Spain and in 1989 in Portugal [1]. Later, phylogenetic studies revealed an independent isolated evolution of the virus with the detection of only G1 strains [17,18,19]. In 2011, the new variant RHDV, described in 2010 in France [13], was reported in Spain [14] and seems to be replacing G1–G5 strains [13,15,16,20,21]. In the Azores, the earliest reports of RHD date back to 1988 in Faial, which is located 9 km from Pico [24]. However, this and other reports of the disease are not conclusive, as the descriptions are minimal. In 2005, the presence of RHDV in Pico was confirmed by the Portuguese Reference Laboratory, but no genetic data were reported.

Despite the patchy information on the history of RHDV in the Azores, our results suggest that the RHDV strains recovered in Pico have evolved independently since the arrival of the virus on this island, becoming a variant of RHDV clearly distinct from all of the other RHDV groups described so far. Nevertheless, the unavailability of samples from previous years prevents us from conducting a phylodynamic analysis that could help to fully determine the origin and relationship of the recovered strains with those that originated other outbreaks in Pico.

A pattern similar to the one observed in Pico was recently observed in Australia, with several RHDV strain lineages having emerged from the originally introduced Czech strain [31]. Indeed, in Australia, the RHDV mutation rate was estimated at 4.0 × 10^−3^ nucleotide substitutions/per nucleotide/per year (with an interval between 3.3–4.7 × 10^−3^). Comparing the Pico sequences with the first G1 and G2 reported in the early 1990s or G3–G5 from 1994 allowed us to estimate a genetic distance of ~0.08; given the mutation rates observed for RHDV in Australia, this would imply a divergence time (or a time to most recent common ancestor) of 20 years with a variance between 24 and 17 years. This scenario is compatible with the appearance of RHDV in Pico between 1989 and 1996, followed by an independent evolution. This would mean that all three main groups (G1, G2 and G3–G5) could have been at the genesis of the RHDV strains detected in Pico. Therefore, the emergence of RHDV in the Azores Islands, an archipelago in the Atlantic Ocean, 1500 km from Europe and Africa, could have occurred by several pathways (birds, trade, tourism) with different origins.

The results show the presence of an RHDV strain in wild rabbits of Pico, Azores, with unique characteristics. Unfortunately, epidemiological information is not available; information on the outbreaks, the mortality in the population and the immune status of the population is scarce. Considering the genetic distance between RHDV Pico and the G1–G5, the former is probably an antigenic subtype of the later, and this means that rabbits infected with G1–G5 have a high probability of being almost fully protected from RHD caused by Pico RHDV. However, the G1–G5 strains seem to have been replaced by RHDV2, both in the wild populations and the domestic breeds [15,16,19,20]. This means that probably in the next few years, European rabbit populations will lose their protection against the G1–G5 RHDV strains.

The *O. cuniculus algirus* only occurs in the southwestern part of the Iberian Peninsula and is a key prey species for several carnivores, including the most endangered feline, the Iberian lynx (*Lynx pardinus*). *O. c. algirus* populations from the mainland are suffering a severe decline due to RHDV [15,20]. Thus, translocations from island populations could be thought of as a solution. Translocations of rabbits from the Azores area to the Iberian mainland should be considered only after a careful study of the main characteristics of this new subtype in order to avoid making worse the already critical condition of the *O. c. algirus* populations, pushing it towards its extinction instead of its recovery. Furthermore, these results show that islands are good models to study viral evolution.
